# New evidence on iron, copper accumulation and zinc depletion and its correlation with DNA integrity in aging human brain regions

**DOI:** 10.4103/0019-5545.64590

**Published:** 2010

**Authors:** P. Vasudevaraju, Bharathi, Jyothsna T, N. M. Shamasundar, K. Subba Rao, B. M. Balaraj, Rao KSJ, Sathyanarayana Rao T.S

**Affiliations:** 1 Department of Biochemistry and Nutrition, Central Food and Technological Research Institute, Mysore -570 020, India; 2 Department of Neurosciences, Institute for Cell Engineering, Johns Hopkins University School of Medicine, Baltimore, Maryland, 21205, USA; 3 Department of Psychiatry, JSS Medical College and Hospital, JSS University, Mysore - 570 004, India; 4 Department of Anatomy, JSS Medical College, JSS University, Mysore, India; 5 Jawaharlal Nehru Technological University, Hyderabad, India; 6 Department of Forensic Science, JSS Medical College, JSS University, Mysore, India

**Keywords:** Aging brain, DNA strand breaks, DNA stability, brain regions, trace metals, oxidative stress

## Abstract

Deoxyribonucleic acid (DNA) conformation and stability play an important role in brain function. Earlier studies reported alterations in DNA integrity in the brain regions of neurological disorders like Parkinson’s and Alzheimer’s diseases. However, there are only limited studies on DNA stability in an aging brain and the factors responsible for genomic instability are still not clear. In this study, we assess the levels of Copper (Cu), Iron (Fe) and Zinc (Zn) in three age groups (Group I: below 40 years), Group II: between 41-60 years) and Group III: above 61 years) in hippocampus and frontal cortex regions of normal brains. The number of samples in each group was eight. Genomic DNA was isolated and DNA integrity was studied by nick translation studies and presented as single and double strand breaks. The number of single strand breaks correspondingly increased with aging compared to double strand breaks. The strand breaks were more in frontal cortex compared to hippocampus. We observed that the levels of Cu and Fe are significantly elevated while Zn is significantly depleted as one progresses from Group I to Group III, indicating changes with aging in frontal cortex and hippocampus. But the elevation of metals was more in frontal cortical region compared to hippocampal region. There was a clear correlation between Cu and Fe levels versus strand breaks in aging brain regions. This indicates that genomic instability is progressive with aging and this will alter the gene expressions. To our knowledge, this is a new comprehensive database to date, looking at the levels of redox metals and corresponding strand breaks in DNA in two brain regions of the aging brain. The biological significance of these findings with relevance to mental health will be discussed.

## INTRODUCTION

The failure in normal healthy aging leads to mental disorders in aged population.[1] Bipolar disorder (BD) is a major geriatric mental health problem. It affects about 1% of the population and causes severe neuropsychological impairments and has been implicated in functional impairment.[2] What we mean by normal and healthy aging and for that matter what are the triggering risk factors for geriatric mental health problems are still puzzling. To understand this better, we need to explore the biology of aging properly. Both structural, chemical, functional brain imaging using magnetic resonance imaging and postmortem studies have demonstrated volume loss in brain in subjects with BD and also with aging.[3–6] Recent postmortem studies in BD have demonstrated reductions in number and density of nerve cells, as well as changes in cell body size and shape of neurons and glia, implicating specific cell pathology in the mood disorders and control aged brains.[4]

These studies give an insight into the central role played by neuronal cell death in the pathology of psychiatric disorders and is absent in normal healthy aging. The major risk factors implicated in age related disorders, is the elevation in oxidative stress and failure in antioxidant mechanisms.[7–12] The oxidative stress phenomenon leads to DNA instability and gene expression failure in normal aging. Does the failure in repair mechanism lead to neuropsychiatric problems? The data base on this aspect is limited. A dysregulation in apoptotic mechanism is believed to play a role in a variety of neuropsychiatric disorders.[13–19] Further, DNA fragmentations have been well shown to be associated with neurodegenerative disorders like Parkinson disease (PD).[20–23] The current study aims to assess the genomic integrity in terms of DNA fragmentation and its relation to the levels of redox active metals in frontal cortex and hippocampal brain regions of different age groups and to ascertain whether altered genome integrity plays a role in geriatric psychiatric disorders. To the best of our knowledge, this study is first of its kind in human brain.

## MATERIAL AND METHODS

**Subjects:** The data covering socio-demographic details and cause of death are given in Table 1.

**Table 1:** Demographic data of aging subjects

Number	Age, yr	PMI, hr	Tissue pH	Sex	Cause of death
Group I<40 yrs					
N-1	25	4	6.76	F	Road traffic accident
N-2	38	5	6.61	F	Snake bite
N-3	21	4	6.71	F	Accidental burns
N-4	18	4	6.45	M	Road traffic accident
N-5	22	5	6.68	M	Road traffic accident
N-6	24	6	6.72	M	Road traffic accident
N-7	30	3	6.54	F	Accidental burns
N-8	37	8	6.45	M	Fall from height
Group II 41-60 yrs					
N-1	45	6	6.67	M	Road traffic accident
N-2	48	5	6.23	F	Road traffic accident
N-3	43	7	6.70	M	Road traffic accident
N-4	55	6	6.51	M	Road traffic accident
N-5	50	6	6.31	F	Road traffic accident
N-6	53	6	6.53	F	Road traffic accident
N-7	51	6	6.32	M	Road traffic accident
N-8	58	6	6.27	M	Road traffic accident
Group III >61 yrs					
N1	65	7	6.77	M	Natural death: collected under body donation to JSS
N2	71	6	6.39	M	Natural death: collected under body donation to JSS
N3	68	7	6.76	M	Natural death: collected under body donation to JSS
N4	77	6	6.57	F	Natural death: collected under body donation to JSS
N5	80	6	6.88	F	Natural death: collected under body donation to JSS
N6	63	6	6.35	F	Road traffic accident
N7	73	7	6.45	M	Road traffic accident
N8	77	6	6.55	M	Road traffic accident

Abbreviations: N, normal; PMI, postmortem interval; SD, standard deviation.

### Materials

Radiolabeled ^3^ [H]-TTP (Sp.Act. 40Ci/nmol) was purchased from Amersham Radiochemicals, UK. Ribonuclease A (RNAse a), Proteinase k, Deoxyribonuclease I (DNAse I), dATP, dTTP, dCTP, dGTP, DNA polymerase I (from *Escherichia coli*), terminal deoxynucleotidyl transferase enzymes, 1 kb and 100 bp DNA ladders, and lamda DNA ladder were purchased from Genei, India. All other chemicals were of analytical grade and purchased from Sisco Research Labs, Mumbai, India.

### Brain tissues

Brains were categorized into three groups. Group I: below 40 years, Group II: between 41-60 years and Group III: above 60 years. The two regions, namely hippocampus and frontal cortex of normal brains, were separated and stored at -80°C until further use. Eight brain samples from each group were included in the study. Human brain samples were collected from the Depression Brain Bank of JSS Medical College and Hospital, Mysore, India. Autopsies were performed on donors with written informed consent obtained direct next of kin. The control human brains were collected from accident victims, who had no history of long-term illness, psychiatric diseases, dementia, or neurological disease prior to death. We have excluded subjects who had drug and alcohol abuse. The average postmortem interval between the time of death and collection of brain and freezing was ≤ six hours. Within one hour after death the body was kept in cool chamber maintained at 4°C. The brain tissue was isolated and stored frozen at -80°C till the analysis.

### Isolation of DNA from brain tissue

Genomic DNA was isolated from hippocampus and frontal cortex of frozen brain tissue by standard ‘phenol-chloroform extraction’ method after Sambrook *et al*.[24] with some modifications to prevent DNA fragmentation during isolation. Precautions were taken to prevent in vitro DNA damage during phenol-chloroform genomic DNA extraction. DNA concentration was measured using ultraviolet/visible spectrophotometer noting absorbance at 260 mm and purity checked by recording the ratio of absorbance at 260 nm/280 nm, which should be ideally between 1.6 and 1.8.

- DNA integrity:
  - Single strand breaks: Single strand breaks (SSBs) are calculated through incorporation of ^3^ [H] -TMP into DNA samples when incubated with *E. coli* DNA polymerase I (Klenow fragment) in a nick translation assay.[25] DNA polymerase I adds nucleotides at the 3’-OH end of a SSB, generated by various means, using the other strand as template. When one of the deoxynucleotide triphosphates is labeled, the incorporation of radioactivity into substrate DNA would be proportional to the number of SSBs present in the DNA sample. During standardization of the assay conditions with the plasmid DNA (Cos T fragment of λ phage) having known number of SSBs, it was found that average of 1500 nucleotides are added at each of the 3’-OH group. From this, it is inferred that each picomole of TMP incorporated is equivalent to 1.6x10^9^ 3’-OH groups or SSBs. In a total reaction volume of 50 *µ*l, the assay mixture consisted of: 40 mM Tris-HC1, pH 8.0, 1 mM β-mercaptoethanol, 7.5 mM MgCl_2_, 4 mM ATP, 100*µ*M each of dATP, dCTP, and dGTP and 25 *µ*M of dTTP, 1 *µ*Ci of ^3^ [H]-TTP and 1*µ*g of genomic DNA and 1 U of *E. coli* DNA polymerase I.
  - **Double strand breaks:** Terminal deoxynucleotidyl transferase catalyzes the addition of deoxynucleotides to the 3’ termini of DNA and does not need direction from template strand. Here, 3’- ends of duplex DNA also serve as substrates. Similar conditions to incubate DNA with terminal transferase as in the case of E. coli polymerase I assay were used. The incorporation of the 3 [H]-dTTP into DNA would be proportional to the number of double strand breaks (DSBs) in the DNA. From the conditions and incubation[26,27] it is assumed that about 50 TMP residues are added at each of the 3’-ends of the duplex DNA. From this, it is calculated that each femtomole of TMP incorporation would be equivalent to 1.2×10^7^ 3’-ends or half of that number minus one DSBs. The assay mixture for terminal transferase reaction consisted of a total volume of 50 *µ*1:100 mM sodium cacodylate buffer, pH 7.0, 1 mM CoCl_2_, 0.2 mM DTT, 1 *µ* Ci of ^3^ [H]-dTTP, 1 *µ*g DNA, and 1 U of the enzyme.
- Trace elemental analysis:
  Brain tissues were acid digested and preserved in dust free laminar flood hood until further use. All the precautions were taken in accordance with National Committee for Clinical Laboratory Standards (NCCLS) criteria (NCCLS standard approved guidelines to eliminate metal contamination while collecting and storing the samples).
- Instrumentation and Elemental Analysis
  Elemental analysis was carried out using Inductively Coupled Plasma – Atomic Emission Spectrometry (ICP-AES) model JOBIN YVON 38 sequential analyzer. The elements measured were Cu, Fe, and Zn. All dilutions were made with ultra pure milliQ water (18-mega ohms resistance) in a dust free environment. The optimization of ICPAES was carried out by line selection and detection limits for each element. The validation of the analysis was tested by analyzing matrix match multi element synthetic standard and certified standard reference material (Bovine liver 1577a) obtained from National Bureau of Standards, USA. The lines were selected for each element in such a way that interference from the other elements was minimal.
  The wavelength used and detection limit of the elements are summarized below:
- Statistical analysis
  All the data obtained in this study were statistically treated and the significance of differences among samples was calculated according to Student’s *t* test. The statistical analysis was carried out using Microsoft Excel 2000 Software.

**Table:** 

Element	Wavelength (nm)	Detection limit*	
		*µ*g/mL (ppm)	*µ*mole/mL
Cu	224.7	0.002	0.00003
Zn	213.856	0.002	0.00003
Fe	259.94	0.005	0.00009

* Detection limit (*µ* g/mL) for each element was calculated by running a multi-element standard solution containing 500 ng/mL of each of the above-cited elements.

## RESULTS

*Trace metals:* The levels of Fe and Cu increased, Zn levels decreased from Group I to III. But the significant increase and decrease was more between Group II and III [Table 2]. This data indicates that there was an accumulation of redox active metals like Cu and Fe with aging, while antioxidant metal was decreased with aging. The interesting point was, accumulation of metals was more in Frontal cortex compared to hippocampus. *This data is novel and first of its kind in literature*.

**Table 2:** Trace metal concentration in two regions of human brains (Concentration in mg/g of wet weight of tissue). Mean ± SD of 8 brains in each group

Brain regions	Trace metals	Group I (N=8)	Group II (N=8)	Group III (N=8)
Fontal Cortex	Cu	4 .0 ± 1.7	5 .0 ± 1.6*	8.0± 1.3**
	Fe	50.8 ±2.6	60.5±3.5*	75±5.6**
	Zn	7.5 ± 0.5	6.5±1.1*	4.5±0.9**
Hippocampus	Cu	4.0 ± 1.0	4.5±1.1*	5.8±1.3*
	Fe	26.6 ±1.9	30.6±1.6*	45±1.75**
	Zn	6.5 ± 0.5	5.5±0.9*	5.0±0.6*

* *P*>0.05,

** *P*>0.001

### Single Strand Breaks

The most prevalent type of DNA damage in mammalian cell is the SSBs. Single-stranded breakage is the end point of several types of structural insults inflicted on the genome by both endogenous and exogenous agents.[28] Table 3 shows numbers of SSBs per microgram of genomic DNA isolated from brain regions. Accumulations of SSBs were more frequent in group III compared to Group II and I. The result shows that frontal cortex (*P*<0.05) accumulated considerably higher number of SSBs compared to hippocampus.

**Table 3:** Single strand breaks (SSBs (10^6^)/µg DNA) in two brain regions of aging brains

Brain regions	Group I (N=8)	Group II (N=8)	Group III (N=8)
Fontal cortex	750	1000*	1750**
Hippocampus	500	600*	850*

* *P*>0.05,

** *P*>0.001

### Double Strand breaks

The DNA isolated from frontal cortex and hippocampus showed significantly higher number of DSBs than respective controls (P<0.01) Table 4. Further, frontal cortex accumulated more DSBs than SSBs. The increase in DSBs was more in Group III compared to Group I and II. The present result showed that frontal cortex has more DSBs than SSBs whereas hippocampus had the presence of both DSBs and SSBs accumulated.

**Table 4:** Double strand breaks (DSBs×106/µgDNA) in two brain regions of aging brains

Brain regions	Group I (N=8)	Group II (N=8)	Group III (N=8)
Fontal cortex	1000	1500*	2750**
Hippocampus	600	720*	950*

* *P*>0.05,

** *P*>0.001

## DISCUSSION

The present study was done to assess the DNA topology in aging human brain and shows that the structural integrity and topology of genomic DNA is altered and also showed a correlation between redox metal accumulation and DNA strand breaks. The DNA integrity failure may lead to cell atrophy. Magnetic Resonance Imaging (MRI) studies showed that frontal cortex, temporal lobe, hippocampus, thalamus and cerebellum are important brain regions in the pathology of mental illness in adults.[3] The previous studies report that smaller cerebellum,[31] reduction in thalamus volume in adolescent[32] and frontal cortex size reduction[33,34] in BD compared to controls. Frontal cortex dysfunction plays a major role in the pathophysiology of the bipolar illness and is correlated with reduced frontal lobe size, neuropsychologic deficits[33,34] and loss of bundle coherence in prefrontal white matter tracts.[35] Other factors like oxidative stress, mitochondrial dysfunction, mitochondrial DNA-deletion, apoptosis are associated with progression of Bipolar mental illness.[9–12,36,37]

The classical apoptotic DNA laddering pattern in aging brain due to strand breaks has great pathophysiological significance. According to Didier *et al*.[38] the DNA laddering on gel electrophoresis is a hallmark of end-stage apoptotic cell death and by this apoptosis can be distinguished from necrosis. Earlier studies have shown that cell death can also be preceded by DNA fragmentation by Ca^2+^ , Mg^2+^ dependent DNAase into 180 and 200bp fragments with endonuclease activation occurring early in the process of cell death.[39–41]

The influences of peri-mortem conditions and of ante-mortem hypoxia on DNA fragmentation in postmortem tissue have been demonstrated in some previous studies.[42] However, we evaluated our results on DNA stability/damage and established that postmortem delay (<7) related DNA damage does not account for the changes in aging brains. It was earlier shown that DNA fragmentation reduces the high activation energy barrier required to induce the conformational and topological changes in DNA.[23] In addition, the recent study showed that there is an empiric link between late-life depression and Alzheimer’s Disease (AD) suggesting that the depression may lead to development of AD in some individuals.[43,44]

Our study is the first report to show that there is a selective increase of single strand and double strand breaks in DNA of normal aging brain. The DNA fragmentation can potentially be triggered through many endogenous and exogenous factors such as trace metals, oxidative stress, mitochondrial dysfunction, apoptosis, decreased antioxidant enzymes, genetic factors etc.[8–11,37] The first and most obvious possibility is that neurons may be exposed to oxidative stress. In addition, the postmortem studies have suggested that GABA cells in the anterior cerebral cortex of BD subjects were vulnerable to oxidative stress.[44] Genes play a central role in the clearance of free radicals generated by mitochondrial oxidation reactions such as glutathione synthase, catalase and superoxide dismutase (SOD)- mediated reactions.[42] This suggests that the accumulation of reactive oxygen species (ROS) associated with the oxidative stress would tend to cause potential damage to DNA, proteins and lipids.[45] However, the intact DNA from hippocampus may represent an adaptive compensation to oxidative stress.

In support of this, other finding suggested that GABAergic cells in hippocampus may be resistant to kainic acid - induced excitotoxicity.[44] Our earlier studies have shown that metals like Fe, Al and Cu are accumulated more in BD and these metals can bind and nick DNA. Many of these insults potentially lead to single strand and double strand breaks in DNA leading to genomic instability.[42]

Another possible reason for accumulated DNA fragmentation in aging brain could be due to decreased antioxidant enzymes such as glutathione synthase, catalase and SOD or decreased DNA repair capacity process in BD. Due to increased oxidative stress and genotoxic stress, the genomic DNA’s structural integrity is under constant threat. Hence, any insufficiency in the machinery to counteract the damage leads to accumulation of DNA breaks. A decline in DNA stability signifies the shift between DNA damage and repair. In conclusion, this study is the first examination of the genome integrity in terms of DNA damage and its relation to redox metals levels in brain regions of aging groups. Further, this early and first study may provide initial insight to elucidate the correlation between the DNA damage and trace metals and its role in mental health.
